# Biochemical characterization of the Arctic char (*Salvelinus alpinus*) ovarian progestin membrane receptor

**DOI:** 10.1186/1477-7827-3-64

**Published:** 2005-11-10

**Authors:** A Håkan Berg, Peter Thomas, Per-Erik Olsson

**Affiliations:** 1 The University of Texas at Austin Marine Science Institute, Port Aransas, Texas, USA; 2 Örebro Life Science Center, Department of Natural Sciences, Örebro University, Sweden

## Abstract

Membrane progestin receptors are involved in oocyte maturation in teleosts. However, the maturation-inducing steroid (MIS) does not appear to be conserved among species and several progestins may fulfill this function. So far, complete biochemical characterization has only been performed on a few species. In the present study we have characterized the membrane progestin receptor in Arctic char (Salvelinus alpinu*s*) and show that the 17,20beta-dihydroxy-4-pregnen-3-one (17,20beta-P) receptor also binds several xenobiotics, thus rendering oocyte maturation sensitive to environmental pollutants. We identified a single class of high affinity (K_d_, 13.8 ± 1.1 nM), low capacity (B_max_, 1.6 ± 0.6 pmol/g ovary) binding sites by saturation and Scatchard analyses. Receptor binding displayed rapid association and dissociation kinetics typical of steroid membrane receptors, with t_1/2 _s of less than 1 minute. The 17,20beta-P binding also displayed tissue specificity with high, saturable, and specific 17,20beta-P binding detected in ovaries, heart and gills while no specific binding was observed in muscle, brain or liver. Changes in 17,20beta-P binding during oocyte maturation were consistent with its identity as the oocyte MIS membrane receptor. Incubation of fully-grown ovarian follicles with gonadotropin induced oocyte maturation, which was accompanied by a five-fold increase in 17,20beta-P receptor binding. In addition, competition studies with a variety of steroids revealed that receptor binding is highly specific for 17,20beta-P, the likely maturation-inducing steroid (MIS) in Arctic char. The relative-binding affinities of all the other progestogens and steroids tested were less than 5% of that of 17,20beta-P for the receptor. Several ortho, para derivatives of DDT also showed weak binding affinity for the 17,20beta-P receptor supporting the hypothesis that xenobiotics may bind steroid receptors on the oocyte's surface and might thereby interfere with oocyte growth and maturation.

## Introduction

Meiosis is arrested at prophase 1 in vertebrate oocytes during their growth phase and a surge in gonadotropin secretion is required to induce the resumption of meiosis and oocyte maturation (OM). It has been demonstrated that gonadotropin (luteinizing hormone, LH) initiates oocyte maturation and ovulation in teleost fish and amphibians by stimulating the production of a maturation inducing substance (MIS) by the ovarian follicles [1]. The MISs have been identified as progesterone in a variety of amphibians and as hydroxylated progestins in teleost fishes [2,3]. Two C21 steroids, 17, 20β-dihydroxy-4-pregnen-3-one (17,20β-P) [4] and 17, 20β, 21-trihydroxy-4-pregnen-3-one (20β-S) [5,6], have been positively identified as the MISs in amago salmon and Atlantic croaker, respectively. While 17,20β-P is the major MIS for salmonids and cyprinids [2], 20β-S has been shown to be the predominant MIS in sciaenids and some other perciform fishes [3].

MIS does not induce OM in amphibians and teleosts by the classical mechanism of steroid action, instead it acts at the cell surface by binding to receptors located on the oocyte plasma membrane [3,7]. Activation of the MIS receptor results in induction of OM via a non-genomic mechanism [8] mediated by G-proteins and second messengers such as cAMP [9,10]. Progestin membrane receptors (mPRs) have been identified and characterized in several amphibian and teleost species [11-15]. Moreover, membrane progestin receptor (mPR) upregulation by gonadotropins during OM has been demonstrated in several teleost species [14,16] and has been associated with development of the ability of oocytes to become responsive to the MIS (oocyte maturational competence, OMC) and complete OM [17]. A two-stage model of the gonadotropic control of OM in teleosts has been proposed based on the results with several teleost species showing that priming of fully grown follicle-enclosed oocytes by gonadotropin is required to induce OMC [18]. Early studies in rainbow trout (*Oncorhynchus mykiss*) showing increased OM in response to the MIS after *in vivo *treatment with pituitary extracts provided an initial indication that priming with pituitary factors is necessary for the development of OMC in salmonids [19]. However, most studies have been conducted with perciform fishes, so that the mechanisms regulating the development of OMC and its timing relative to other processes during OM remain poorly understood in other teleosts.

The present study describes a comprehensive characterization of the ovarian mPR in a salmonid, Arctic char (*Salvelinus alpinus*). The Arctic char is of great commercial value in countries in the northern hemisphere, mainly due to its ability to grow at low temperatures, but the species also displays high sensitivity to environmental change. Functional reproduction is of outmost importance for species survival and successful breading in fish farms. Thus it is of great importance to obtain information on the basic mechanisms of reproduction in this species. We demonstrate that the receptor is upregulated during OM. The mPR abundance increase during OM, suggests its involvement in the development of OMC in salmonid fishes. Furthermore, the receptor display binding to xenobiotics, indicating that it is a target for endocrine disruptors that through binding to the mPR may interfere with oocyte maturation and thereby disrupt reproduction.

## Materials and methods

### Chemicals

[1,2,6,7-^3^H]17α-hydroxyprogesterone (specific activity 97 Ci/mmol) was obtained from New England Nuclear (Boston, MA) and enzymatically converted to 17,20β-P as described by [20]. The unlabeled steroids were purchased from either Steraloids, Inc. (Wilton, NH) or from Sigma Chemical Company (St. Louis, MO). The xenobiotics *o*,p'-DDT, *o*,p'-DDE, *o*,p'-DDD, Kepone and methoxychlor were obtained from Chem Services (Westchester, PA). 4-Nonylphenol was obtained from the Huntsman Corporation (Port Neches, TX). Flutamide and cimetidine were purchased from Sigma (St Louis, MO). The antiprogestin ORG31710 was a generous gift of Organon (Oss, The Netherlands) while ZK98,299 and ZK112,993 were generous gifts of Schering AG (Berlin, Germany). All steroids, antihormones and xenobiotics were dissolved in 95% ethanol to appropriate concentrations and stored at -20°C. Chemicals and salts used for making the buffers were purchased from Sigma Chemical Company (St Louis, MO) and from Fisher Scientific (Pittsburgh, PA). The scintillation cocktail was prepared without methanol according to [21].

### Animals

Adult female Arctic char were obtained from Fiskeriverkets Forskningsstation, Kälarne, Sweden and held in indoor tanks with a continuous flow-through water system and fed a commercial Arctic char pelleted diet (Skretting, Norway) daily. A total of 30 fish were used in the present study. Temperature and photoperiod were adjusted to mimic natural conditions during the period of ovarian recrudescence. Fish were maintained under these conditions until their oocytes were fully grown and had diameters of approximately 5 mm.

### Tissue sampling and preparation

Fish were anesthetized with MS222 (Sigma Chemical Company, St Louis, MO), sacrificed, and the ovaries were removed and immediately frozen in liquid nitrogen and stored at -80°C for up to 6 months prior to analysis. Thawed ovarian tissue (1–2 g) was placed between two 700 nm nylon mesh screens and clamped between two boards to rupture the oocytes and expel the yolk. The yolk was subsequently rinsed from the remaining tissue twice with 20 ml of ice-cold HAEW buffer (25 mM Hepes, 1 mM NaCl, 1 mM EDTA, pH 7.4). The tissue fragments were weighed and placed in 15 ml (~1:15 w/v) of ice-cold HAEP buffer (25 mM Hepes, 10 mM dithiothreitol (DTT), 1 mM NaCl, 1 mM EDTA, 1 mM PMSF, pH 7.4) and homogenized using a Polytron (Tekmar Tissuemizer). Homogenization was performed in two steps: first at low speed (setting 4) for 10 seconds and then at moderate speed (setting 7) for 30 seconds. The homogenate was centrifuged at 3,000 g for 5 minutes to remove the nuclear and heavy mitochondrial fractions [22]. The lipid layer was removed from the top and the supernatant was transferred to a new tube. The supernatant was centrifuged at 20,000 g for 5 minutes to pellet the plasma membrane fraction. The final pellet was resuspended in 10 ml of HAEP buffer and stored at -80°C until analysis.

### Receptor Binding Assay

The filtration assay method originally developed by Patiňo and Thomas [12] to measure the mPR in spotted seatrout ovarian membranes was used with minor modifications. Radiolabeled 17,20β-P (final concentration 1 – 20 nM) was dissolved in HAEP buffer and a 250 μl aliquot was added to each assay tube with or without a 100-fold excess of cold steroid. The cold steroids were dissolved in ethanol, added to the assay tubes and the ethanol was dried down under N_2_. To each tube, an aliquot (250 μl) of the membrane preparation was added, the tubes were vortexed and incubated for 30 minutes at +4°C. The binding reaction was stopped by taking a 250 μl aliquot from each tube and passing it through a presoaked glass microfiber filter (Whatman GF/B, 2.1 cm, Whatman, Denmark) on a microfilter holder (Hoefer Scientific Instruments; FH124) attached to a vacuum pump. Each filter was rinsed with 12.5 ml of HAEW buffer and then placed in a 7 ml scintillation vial. Scintillation cocktail (5 ml) was added to each tube, the tubes were shaken for 5 minutes and the radioactivity was measured in a Beckman LS 6000SC scintillation counter (Beckman Instruments, Fullerton, CA). Each sample was assayed in triplicate. Specific binding in each sample was calculated by deducting the non-specific binding from the total binding.

### Saturation analysis

Radiolabeled 17,20β-P (0.625–30 nM) was added to each reaction tube with or without 3 μM cold steroid. Membrane samples were incubated with steroids for 30 minutes at +4°C. The reactions were terminated by filtration and the radioactivity in the filter was determined as described above. Non-linear curve fitting procedures (GraphPad Prism, version 3.03, GraphPad Software Inc) were used to estimate the concentration of 17,20β-P binding sites (B_max_) and to calculate the dissociation constant (K_d_). To determine the time necessary to reach binding equilibrium, membrane preparations were incubated in 7 nM radiolabeled 17,20β-P with or without 700 nM unlabeled 17,20β-P. The reaction was terminated at different time-points ranging between 15 seconds to 4 hours. The specific binding for each time-point was determined as described above.

Dissociation kinetics for [^3^H]-17,20β-P binding to the receptor was determined using standard procedures. Ovarian membrane preparations were first incubated with 7 nM radiolabeled 17,20β-P in the absence (total binding) or presence (nonspecific binding) of 700 nM unlabeled 17,20β-P for 30 minutes to ensure maximum association of the radioligand to the receptor. Aliquots (500 μl) of the membrane receptor suspensions were subsequently added to tubes containing 700 nM unlabeled 17,20β-P (500 μl) and incubated at +4°C. At various time points ranging between 15 seconds and 4 hours the reactions were terminated and the specific binding for each time point was determined as described above.

### Tissue specificity

Plasma membrane suspensions were made from ovaries, heart, gill and muscle tissue as described above. All samples were assayed in triplicate and the resuspended membrane preparations were incubated with 7 nM of radiolabeled 17,20β-P in the presence or absence of 700 nM unlabeled steroid. After 30 minutes incubation at +4°C the reaction was terminated by filtration and the specific binding for each membrane preparation was determined as described above.

### Steroid specificity

The steroid specificity of receptor binding was examined using a competitive binding assay. Ovarian tissue membrane preparations were incubated for 30 minutes in tubes containing 7 nM [^3^H]-17,20β-P with or without 11 different steroids at concentrations ranging between 0.1 nM and 10 μM. The following steroids were tested: 17,20β-P, 20β-S, progesterone (P4), cortisol (F), 17β-estradiol (E2), 11-ketotestosterone (11-KT), testosterone (T), 11-deoxycorticosterone (11-DOC), 17α-hydroxyprogesterone (17α-OH-P), 11-deoxycortisol (DC), and pregnenolone (P5). All cold steroids were dissolved in ethanol and added to the assay tubes. The ethanol was subsequently dried down under N_2_. After 30 minutes incubation at +4°C the reaction was terminated by filtration and the specific binding was determined as described above.

### Effects of *in vitro *gonadotropin treatment on 17,20β-P binding to ovarian mPR

The possible hormonal regulation of ovarian 17,20β-P receptor by gonadotropin was investigated *in vitro*. Freshly prepared ovarian fragments (3 g) were incubated in Dulbecco's Modified Eagle's medium with Ham's nutrient mixture F-12 (DME/F12) (30 ml) containing 1, 7 and 14 IU human chorionic gonadotropin (hCG)/ml. The fragments were incubated at +15°C for 20 hours under an atmosphere of oxygen. At the end of the incubation the medium was discarded and the tissue fragments stored at -80°C until assayed for 17,20β-P binding.

To investigate the time course of 17,20β-P receptor regulation by hCG, ovarian fragments (3 g) were incubated for time-points ranging between 0 and 20 hours in DME/F12 containing 14 IU hCG/ml. At the end of each incubation the ovarian fragments were removed from the medium, some of the oocytes were examined for germinal vesicle migration (GVM) and germinal vesicle breakdown (GVBD), and the remaining ovarian tissue was immediately stored at -80°C until analysis. Estimations of 17,20β-P binding were obtained as described above.

### Interactions of xenobiotics and hormone antagonists with the mPR

The binding affinities of a variety of xenobiotic chemicals for the 17,20β-P membrane receptor were investigated to determine whether the 17,20β-P action mediated by this receptor was potentially susceptible to chemical disruption. The xenoestrogens nonylphenol, Kepone, and o,p'-DDT with its metabolites o,p'-DDE and o,p'-DDD, the androgen antagonist flutamide, the progestin antagonists ZK 98299, ZK112992 and ORG 31710 and the H2 histamine receptor antagonist cimetidine were tested for receptor binding. All xenobiotics were added to the assay tubes dissolved in ethanol, which was dried down under N_2 _prior to addition of tissue preparation. The ovarian tissue suspensions were prepared as described above and incubated with 7 nM [^3^H]-17,20β-P in absence or presence of the xenobiotics over a broad range of concentrations (0,1 nM – 1 mM). After a 30 minutes incubation at +4°C free steroid was removed by filtration. The specific binding for each tissue suspension was determined as described above. The binding of xenobiotics to the 17,20β-P receptor was expressed as a percentage of the maximum specific binding of 17,20β-P binding to its receptor.

### Data analysis

The significance (P) was calculated using a one way ANOVA followed by Bonferroni's multiple comparison test with a P < 0.05. All statistical analysis was performed using GraphPad Prism version 3.03 for Windows (GraphPad Software, San Diego California USA).

## Results

### Receptor characterization

**Figure 1:**
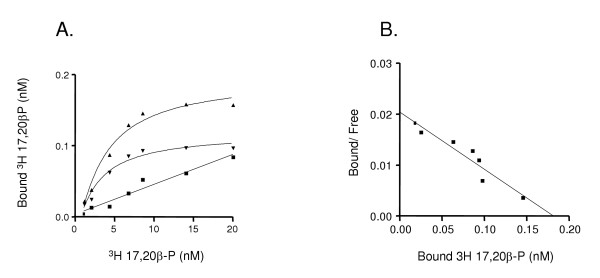
**Representative saturation curve (A) and Scatchard analysis (B)**. Specific binding of [^3^H]-17,20β-P to the plasma membrane fraction of Arctic char ovaries using membrane suspensions incubated with [^3^H]-17,20β-P at concentrations ranging between 1 nM – 20 nM with or without a 100 fold excess non radiolabeled 17,20β-P. Specific binding (▼) was determined by subtracting non-specific binding (■) from total binding (▲). TB: total binding, NSB: nonspecific binding, SB: specific binding. All points are means of triplicate determinations.

High affinity (Kd, 13.8 ± 1.1 nM), specific 17,20β-P binding sites were detected on Arctic char ovarian plasma membranes by saturation analysis. Receptor binding was saturated at a concentration of 7 nM 17,20β-P and Scatchard analysis showed a single class of limited capacity (Bmax, 1.6 ± 0.6 pmol/mg ovary) binding sites for 17,20β-P (Fig. 1). Specific binding was detected over the pH range of 7.0 to 7.8 with maximum receptor binding at pH 7.4 (data not shown).

**Figure 2:**
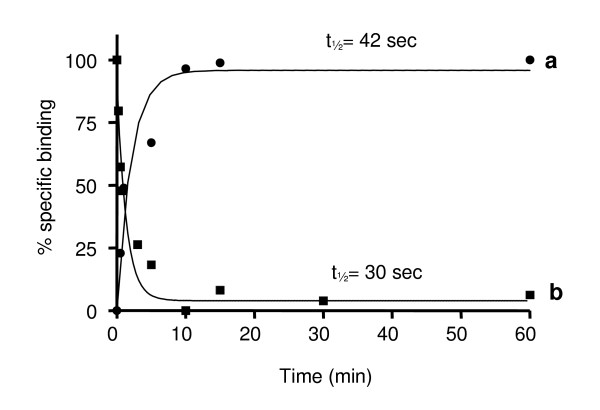
**Association (●) and dissociation (■) kinetics**. [^3^H]-17,20β-P binding to Arctic char ovarian plasma membrane preparations. The reactions were terminated at time-points ranging between 15 seconds and 4 hours. The data are expressed as percentages of maximum specific binding. Each point represents the average of three replicate assays.

The association kinetics of 17,20β-P binding to the receptor was rapid and saturation of the binding sites was achieved after 3 minutes at +4°C, with a t_1/2 _of 42 ± 3.4 seconds (Fig. 2). The non-specific binding did not change throughout the incubation time. The binding of [^3^H]-17,20β-P to the receptor was readily displaced with unlabeled 17,20β-P. The kinetics of dissociation were rapid and [^3^H]-17,20β-P was completely dissociated from the receptor after 5 minutes of incubation at +4°C, with a t_1/2 _of 30 ± 4.2 seconds (Fig. 2).

**Figure 3:**
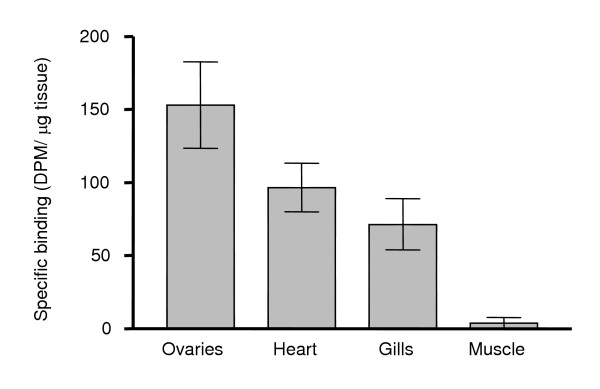
**Relative receptor abundance in different tissues**. Specific [^3^H]-17,20β-P binding to membrane preparations of Arctic char ovary, heart, gill and muscle tissues. Data are presented as means ± SEM, obtained from three females.

**Figure 4:**
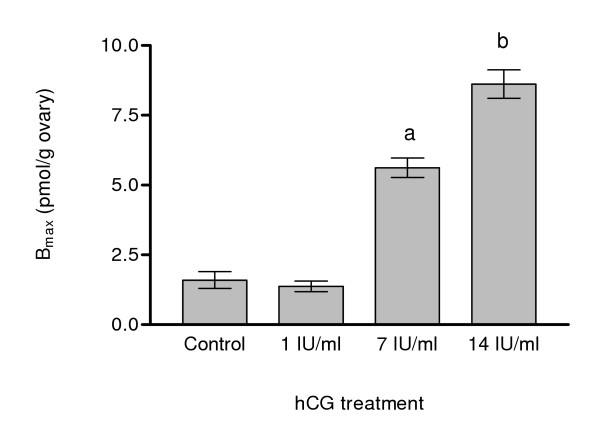
**Concentration-dependent effects of in vitro gonadotropin (hCG) treatment on ovarian 17,20β-P binding to mPR**. Ovarian fragments were incubated with 0, 1, 7, or 14 IU hCG for 20 hours. Each bar represents the mean ± SEM of triplicate determinations. Significant difference at the p < 0.01 level compared to control (a) and the 7 IU group (b) are indicated in the figure.

**Figure 5:**
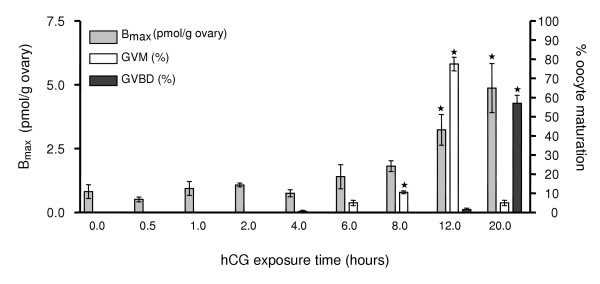
**Time course of increased ovarian 17,20β-P binding to mPR and oocyte maturation**. Arctic char ovarian fragments were incubated with 14 IU hCG/ml. At the end of each incubation period, oocytes were examined for 17,20β-P binding and the occurrence of GVM and GVBD. Each bar represents the mean ± SEM from 5 different fishes, each one assayed in triplicate. * P < 0.01 compared to other time points.

Specific 17,20β-P binding was detected in membrane fractions of ovary, heart and gill Arctic char tissues, whereas no binding was detected in muscle tissue (Fig. 3). Highest 17,20β-P receptor binding was measured in ovarian tissue. High amounts of 17,20β-P binding were also observed in brain and liver tissues but subsequent saturation and Scatchard analyses showed that these binding moieties did not have the binding characteristics of steroid receptors (data not shown). *In vitro *treatment of ovarian fragments with gonadotropin (hCG) for 20 hours caused a concentration-dependent increase in 17,20β-P binding to the mPR. Maximum induction of the mPR, a five-fold increase over initial levels, was observed following treatment with 14 IU hCG/ml (Fig. 4). The time course study revealed that 17,20β-P binding to the mPR increased after 6–8 h of hCG treatment (Fig. 5), but that the increase was not significant until 12 hours of hCG treatment. Maximum receptor binding was not observed until after 20 hours of hCG exposure. These changes in mPR binding were accompanied by the induction of maturation of the follicle-enclosed oocytes. GVM, an early stage of OM, was first observed in about 10% of the oocytes after 6 hours of hCG incubation (Fig. 5). The proportion of oocytes undergoing GVM reached a maximum of 75% after 12 hours and subsequently declined at 20 hours as the oocytes proceeded to the later GVBD stage of OM.

**Figure 6:**
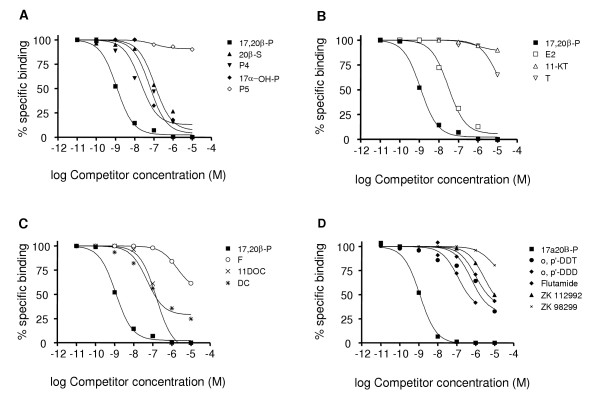
**Competition by various steroids for binding of [^3^H]17,20β-P to ovarian plasma membranes**. Ovarian membrane preparations were incubated for 30 minutes with [^3^H]-17,20β-P in the presence of 10 pM – 10 μM competitor. For explanation of abbreviations see Materials and Methods. Each point represents the mean of triplicate determinations. **A**) progestogens. **B) **androgens and estrogen. **C) **corticosteroids. **D) **xenobiotics.

Competition studies showed that steroid binding to the Arctic char ovarian membrane fraction was specific for C21 Δ4 (4-ene) steroids lacking a functional group at the 11 position, cortisol and pregnenolone displaying negligible affinity for the receptor (Fig. 6a,c). The common fish androgens (C19 steroids) T and 11-KT also showed little or no affinity for the receptor (Fig. 6b). The C18 steroid E2 displayed the highest relative binding affinity (5%) to the receptor of the other major steroids present in fish plasma (Table 1). 17,20β-P, the MIS in salmonids, showed 20× higher binding affinity for the receptor than any of the other steroids or xenobiotics tested. Removal of the OH group at the 20 position of the side chain of 17,20β-P, resulting in the formation of 17αOH-P, resulted in a forty-fold decrease in the relative binding activity (RBA) which was not further altered by the additional removal of the OH group on the 17 position (P4). Addition of an OH group at the 21 position of 17,20β-P, to produce 20β-S, the other major fish MIS, caused an 80 fold decline in binding affinity. Other 11-deoxycorticosteroids (11-DOC, DC) had similar RBAs as 20β-S. The presence of a functional (OH) group at the 11 position of a C21 steroid, cortisol (F), resulted in a further loss of binding affinity. Finally alterations of the ketone at the 3 position of progesterone to a hydroxyl and repositioning of the double bond to the 5 position (P5, Δ5, 5-ene steroid) resulted in a complete loss of binding affinity.

**Table 1:** EC 50 and relative binding affinity (RBA) of various steroids and xenobiotics for Arctic char ovarian membrane fractions.

**Competitor**	**Concentration (nM) causing 50% displacement**	**RBA (%)**
17,20β-P	1.25	100.0
E2	37.9	4.68
P4	52.6	3.37
17α-OH-P	69.3	2.56
11-DOC	132.3	1.34
20β-S	145.2	1.22
DC	207.1	0.86
Flutamide	256.1	0.49
o,p'-DDT	377.5	0.37
o,p'-DDD	549.2	0.30
ZK 112992	1179	0.11
ZK 98299	12879.8	0.01
F	14421.2	0.01
T	18347.21	0.01
11-KT	ND	0.0
P5	ND	0.0
Kepone	ND	0.0
Nonylphenol	ND	0.0
o,p'-DDE	ND	0.0
Cimetidine	ND	0.0
ORG 31710	ND	0.0

ND indicates substances that did not display 50% displacement of 17,20β-P from its receptor in the range of concentrations examined (steroids: 0.1 nM – 10μM, RBA < 0.01%; xenobiotics, hormone antagonists: 0.1 nM – 1 mM, RBA < 0.0001%).

All synthetic hormone antagonists and xenobiotics tested showed low or no binding affinity for the 17,20β-P receptor (Table 1; Fig. 6d). The highest binding affinity was displayed by the androgen antagonist flutamide, which had a RBA of 0.49%, a 50× higher affinity than T, the strongest binding androgen. The progestin antagonist ZK112992 also showed weak binding affinity (0.11%) for the mPR. Among the xenobiotics tested o,p'-DDT and its metabolite o,p'-DDD had a RBA less than 1% that of the natural ligand, 17,20β-P (0.37% and 0.30% respectively), while the other DDT metabolite o,p'-DDE, Kepone and nonylphenol did not bind the 17,20β-P receptor.

## Discussion

In the present study, a 17,20β-P binding moiety was identified in Arctic char ovarian plasma membranes that fulfil all the criteria for its designation as a steroid membrane receptor. A single class of high affinity (K_d_, 13.8 ± 1.13 nM, N = 6), saturable (B_max_, 1.6 ± 0.6 pmol/g ovary) and displaceable, specific 17,20β-P binding sites are detectable on ovarian membranes. The kinetics of 17,20β-P association and dissociation to the membrane binding sites are very rapid, with t_1/2 _s less then a minute, which is typical of steroid membrane receptors. The binding is highly specific for 17,20β-P and other progestins and is negligible for other steroids. The binding also shows tissue specificity, with highest levels of specific binding in the ovaries, a known target of 17,20β-P action. It was also found that 17,20β-P binding to mPR in Arctic char ovaries increase dramatically during OM which supports the suggestion that gonadotropin-induced upregulation of the MIS receptor is a common occurrence amongst teleosts and is of physiological importance during OM [17].

Specific binding of the salmonid MIS, 17,20β-P, and the synthetic progestin R5020 has been demonstrated with ovarian and oocyte plasma membranes from brook trout (*Salvelinus fontinalis*) [23] and rainbow trout [13]. Overall, the binding characteristics of the Arctic char ovarian mPR are similar to those reported previously. The affinity of 17,20β-P binding (K_d_, 13.8 nM, to the mPR on Arctic char ovaries is similar to that reported for MIS binding to the mPR in rainbow trout (*Oncorhynchus mykiss*, K_d_-18 nM) and yellowtail (*Seriola quinqueradiata *K_d_, 22.9 nM), but somewhat lower than that reported in spotted seatrout (*Cynoscion nebulosus*, ovaries: K_d_, 1.5–6.0 nM) and striped bass (*Morone saxatilis *K_d_, 1.4 nM) gonadal membrane fractions [12-15]. The slightly higher K_d_s in rainbow trout tissues could be related to the higher amounts of MIS present in the plasma of this species [24] compared with Arctic char [25], while the lower K_d _found in spotted seatrout and striped bass correlates well with lower MIS plasma levels during OM in these species [17,26].

The Arctic char ovarian mPR has a limited binding capacity for 17,20β-P (1.6 ± 0.6 pmol/g ovary), similar to the 17,20β-P receptor binding capacity reported previously in yellowtail (2.1 pmol/g ovary; [15]). The membrane receptor demonstrated rapid association and dissociation kinetics to the ligand as well as high ligand and tissue specificity, all specific characteristics of a steroid membrane receptor. The association and dissociation rates (T_1/2 _< 1 minute, equilibrium reached within 3 minutes) were similar to previously reported ovarian membrane receptor binding for MIS in some sciaenids [12], but differs from the reported t_1/2 _from carangids [15] where the t_1/2 _s were found to be slower (15 minutes).

An interesting finding was that gonadotropin induction of OM *in vitro *was associated with a dramatic five-fold increase in mPR concentrations in Arctic char ovarian tissues. Upregulation of mPR concentrations by gonadotropin during induction of OM *in vitro *has been observed previously in spotted seatrout ovaries [17,27]. It has been suggested that exposure to gonadotropin is necessary for the development of oocyte maturational competence and the onset of OM [28]. The Arctic char 17,20β-P membrane receptor displayed dose-dependent induction when preincubated in hCG for 20 hours. A time-course experiment showed that 17,20β-P binding activity was significantly increased after 12 hours treatment with hCG. The timing of the mPR upregulation was associated with GVM and the onset of GVBD after gonadotropin treatment. GVM occurred in a high percentage of the oocytes after 12 hours, and GVBD was observed between 12 and 20 hours of hCG treatment of whole Arctic char ovary fragments. These results are consistent with a physiological role for increased 17,20β-P binding to the mPR on the oocyte surface during oocyte maturation.

Determination of steroid specificity showed that 17,20β-P had the highest binding affinity, followed by E2 (4.68%) while 11-DOC and 20 β-S only had RBAs of 1.34% and 1.22%, respectively. These RBAs are comparable to results obtained with the yellowtail mPR, where 17,20β-P is the ligand with highest affinity [15]. In yellowtail the receptor binding-affinity for 11-DOC was slightly higher (3.2%) and 20β-S was slightly lower (0.8%) than observed for Arctic char.

It has been found that xenobiotic chemicals can interfere with nongenomic, cell surface-mediated actions of steroids [29,30]. Low, environmentally realistic, concentrations of a variety of xenobiotics has been shown to impair OM *in vitro *in response to the MIS in spotted seatrout and Atlantic croaker ovarian fragments [29,31], and also antagonize MIS upregulation of sperm motility [32]. The observation that these compounds could bind to the mPR on seatrout oocytes and croaker sperm provided the first indication that chemicals could interfere with nongenomic steroid actions by a receptor-mediated mechanism. Xenobiotic chemical binding to mPR was confirmed in a salmonid species for several of these compounds in the present study. In Arctic char both o,p'DDT and o,p'DDD showed significant binding to the mPR, with RBAs of 0.37 ± 0.04% and 0.30 ± 0.04% respectively. However, Kepone and the other xenobiotics tested did not show measurable binding to the membrane 17,20β-P receptor at concentrations up to 1 mM. In contrast, Kepone displayed the highest binding affinity of the chemicals tested for the spotted seatrout mPR (RBA ~ 0.1% relative to 20β-S), followed by o,p'DDD, methoxychlor and o,p'-DDT which showed weak displacement of 20β-S binding [29]. These differences in the binding affinities of xenobiotics for the spotted seatrout and Arctic char mPRs are not surprising, since these receptors also display marked differences in their RBAs for steroids. The spotted seatrout mPR shows the highest binding affinity for 20β-S and 17,20β-P has a RBA of 0.64% [32], while Arctic char mPR shows highest binding affinity for 17,20β-P and 20β-S has a RBA of 1.2%. These results indicate that the mPR located on Arctic char ovarian plasma membranes is a possible target for endocrine disruption. However, further research is needed to determine the extent of xenobiotic interactions with mPRs and their effects on reproduction.

In conclusion, this study shows the presence of a highly specific 17,20β-P receptor located in the ovarian plasma membrane in Arctic char. The receptor was identified as a high affinity and a low capacity mPR. This study also suggests that abundance of the Arctic char mPR has biological relevance since both the receptor density on the oocyte surface and the onset of OM clearly are regulated by gonadotropin. Furthermore, the interaction of xenobiotics with mPR suggests that it may be a target for endocrine disruption resulting in reduced OM thereby reducing the reproductive success.
